# MeSAUR1, Encoded by a Small Auxin-Up RNA Gene, Acts as a Transcription Regulator to Positively Regulate *ADP-Glucose Pyrophosphorylase Small Subunit1a* Gene in Cassava

**DOI:** 10.3389/fpls.2017.01315

**Published:** 2017-07-31

**Authors:** Ping’an Ma, Xin Chen, Chen Liu, Yuhong Meng, Zhiqiang Xia, Changying Zeng, Cheng Lu, Wenquan Wang

**Affiliations:** ^1^Institute of Tropical Agriculture and Forestry, Hainan University Haikou, China; ^2^The Institute of Tropical Bioscience and Biotechnology, Chinese Academy of Tropical Agricultural Sciences Haikou, China; ^3^Key Laboratory of Biology and Genetic Resources of Tropical Crops, Ministry of Agriculture Haikou, China

**Keywords:** cassava, small auxin-up RNA gene, ADP-glucose pyrophosphorylase, starch biosynthesis, transcription factor

## Abstract

Cassava, being one of the top three tuberous crops, features highly efficient starch accumulation in the storage root to adapt the tropical resources and environments. The molecular mechanism for the process, however, is still unclear. ADP-glucose pyrophosphorylase, the first and rate-limited enzyme in starch biosynthesis pathway, is a heterotetramer comprised of two small/catalytic and two large/modulatory subunits. To understand the regulation of *MeAGPase*, the promoter of a highly expressed small subunit, *MeAGPs1a*, was used as bait for a yeast one-hybrid assay to screen storage root cDNA library. One cDNA, coding for a small auxin-up RNA protein, named *MeSAUR1*, was isolated from cassava. MeSAUR1 could bind to the promoter of *MeAGPS1a* in yeast one-hybrid test and *in vitro*, and was located in cell nucleus. *MeSAUR1* displayed a higher transcript level in cassava root cortex, and its expression was induced by indole-3-acetic acid, gibberellin and ethylene, but repressed by abscisic acid. A dual-luciferase interaction test further convinced that MeSAUR1 could bind to the promoter of *MeAGPS1a*, and positively regulate the transcription of *MeAGPS1a* in cassava.

## Introduction

Cassava (*Manihot esculenta* Crantz), originated from Amazon tropical rainforest in South America, is now one of the top three tuberous crops in the global world. Cassava storage root/starch is not only the main staple for African and South American people, but also an important source for food processing and bio-energy industry. Starch is an insoluble polymer of glucose residues produced by the majority of higher plant species, and mainly stored in seeds and storage organs (Tetlow et al., 2004). ADP-glucose pyrophosphorylase (AGPase) is a rate-limited enzyme by utilizing ADP-glucose as the glucosyl donor for the elongation of α-1,4-glucosidic chains in starch biosynthesis pathway, and it consists of two small/catalytic and two large/modulatory subunits as a heterotetramer in higher plants (Ballicora et al., 2004; Saripalli and Gupta, 2015). The AGPase gene family has been characterized in several higher plants, e.g., two small subunits (SS) and several large subunits (LS) in rice and sweet potato, respectively (Akihiro et al., 2005; Zhou et al., 2016). Over-expressing SS gene or/and LS gene could enhance AGPase activity, increase seed weight and starch content (Li et al., 2011).

Auxin is an important plant hormone which can regulate cell elongation and division, root development and the formation of vascular bundle, flower and other organs. In plant, the Auxin responsive genes are mainly divided into three groups: auxin/indole-3-acetic acid (*Aux/IAA*), gretchenhagen-3 (*GH3*) and small auxin-up RNA (*SAUR*). The *SAUR* genes are the largest auxin responsive gene family, normally with the characteristics of no intron, and one or several auxin responsive elements in their promoter regions (Hagen and Guilfoyle, 2002). SAUR proteins are generally located in plasma membrane, cytoplasm and nucleus, and over-expressing *SAUR* genes could cause the phenotypic changes in leaf size, hypocotyl and root length, and distortion of inflorescence (Chae et al., 2012; Hou et al., 2013; Stamm and Kumar, 2013; Spartz et al., 2017). Moreover, SAUR proteins, e.g., OsSAUR39 and OsSAUR45, also acted as a negative regulator of auxin synthesis and transport in rice, possibly through the repression of *OsYUCCA* and *OsPIN* gene expression (Kant et al., 2009; Xu et al., 2017).

In this study, an Auxin responsive factor gene from cassava, named *MeSAUR1*, was identified through a yeast one-hybrid screening by using the promoter of *MeAGPS1a* as bait and the cDNA expression library of storage root as prey. The MeSAUR1 protein could bind to the bait sequence in yeast and their interaction was also confirmed *in vitro*. Furthermore, a dual-luciferase reporter assay demonstrated that MeSAUR1 could up-regulate the promoter activity of *MeAGPS1a*, suggesting that MeSAUR1 protein might be a positive regulator of *MeAGPS1a* and involved in cassava starch biosynthesis.

## Materials and Methods

### Plant Material

*Manihot esculenta* Crantz cv.KU50 was used for construction of full-length cDNA library and gene expression analysis. The plants were cultivated in the experimental station of the Institute of Tropical Biosciences and Biotechnology, Chinese Academy of Tropical Agricultural Sciences (Chengmai County, Hainan Province, China). Eight tissues, including tip and mature leaves, flowers, petiole, stems rind, root stele, root cortex, tuber roots and primal roots were collected at 180 days after planting. Tissue cultured plantlets of KU50 were grown in MS medium, and the culture condition was 25°C and 16/8 h in light/dark photoperiod. One-month-old plantlets were treated with 100 μM abscisic acid (ABA), or 10 mM Ethephon (2-chloroethyl phosphoric acid, ET), or 100 μM Gibberellin A3 (GA3) or 10 μM 3-Indole acetic acid (IAA, Sigma, United States). Leaves were harvested at 1, 3, 6, and 12 h after hormone treatments, respectively. As control, plantlets were collected from 0 h and each sample included nine plantlets. All materials were immediately stored at -80°C after liquid nitrogen freeze.

### Yeast One-Hybrid

Yeast One-Hybrid assay was conducted according to the method described by Li et al. (2016). The *MeAGPS1a* promoter (1025 bp) was amplified by PCR with V1 primer pair (**Table 1**), and then ligated into the pAbAi vector (Clontech, United States) via the *Hind* III and *Sal* I restriction sites, constructed yeast one-hybrid bait vector pAbAi-MeAGPS1a. Cassava storage root total RNA, samples from root early development, enlargement and mature stages, was extracted using plant RNeasy extraction kit (Tiangen, CHN). The mRNA was purified using the NucleoTrap mRNA Mini Kit (Macherey Nage, GER) and the single-strand cDNA was synthesized using the RevertAid First-Strand cDNA Synthesis Kit (Fermentas, GER) following the steps provided by manufacturer. The double-stranded cDNA was amplified by long-distance PCR and size selected (>200 bp) using Chroma Spin^TM^ TE-400 columns (Clontech, United States). Cassava double-stranded cDNAs, the prey vector pGADT7-Rec with *Sma* I-linearized and the bait vector pAbAi-MeAGPS1a were transformed into the yeast strain Y1HGold (Clontech, United States), then cultivated on SD/-Leu medium with 300 ng/ml Aureobasidin A (AbA) at 30°C for 3 days. The drug-resistant colonies were identified by plasmid PCR and sequence analysis.

**Table 1:** The primer pairs used in this study.

Primer name	Forward primer (5′–3′)	Reverse primer (5′–3′)
V1	AAGCTTCAGCTGCCCCTAC	GTCGACTAGCAAGTTCAGATTTGG
V2	CATATGGCGATCAGGAAATCAAC	GTCGACTCTGAGCATGGATG
V3	GGATCCATGGCGATCAGGA	GTCGACTCTGAGCATGGATG
V4	GAATTCCAGCTGCCCCTACC	ACTAGTTAGCAAGTTCAGATTTGGAAAA
seg5	AAGCTTCAGCTGCCCCT	GTCGACCTAGATAATTTTTAAAATTAATAAATTTTA
seg4	AAGCTTCTAGTAATATGGTATTATAATTAAGG	GTCGACTAAAGTGAAGAGACG
seg3	AAGCTTCATGATGGTTGAAACA	GTCGACTTCTTTTTAGTAAACAAAAGA
seg2	AAGCTTAATACTGACGGTCCTA	GTCGACAGTGTGATTGGGG
seg1	AAGCTTCCGTCCCTCCAA	GTCGACTAGCAAGTTCAGATTT
Biotin-seg2	Biotin-AATACTGACGGTCCTAAAAATCTTAG	Biotin-AGTGTGATTGGGGCAGGG
Biotin-AGPS1a	Biotin-CAGCTGCCCCTACCGTTAA	Biotin-TAGCAAGTTCAGATTTGGAAAAAACC
*MeSAUR1*	GGCGATCAGGAAATCAACCAA	GGACAATGTACCTGCTTCTGTT
β*-actin*	CAAGGGCAACATATGCAAGC	CCTTCGTCTGGACCTTGCTG


In order to confirm the interaction between MeSAUR1 and *MeAGPS1a* promoter, we amplified *MeSAUR1* coding sequence (CDS) with V2 primer pair (**Table 1**). The CDS was ligated into pGADT7 vector through *Nde* I and *Sal* I, and named pGADT7-MeSAUR1. pGADT7-MeSAUR1 and pAbAi-MeAGPS1a were co-transformed into the Y1HGold yeast strain, pGADT7-Rec53+p53-AbAi, pAbAi-MeAGPS1a, pGADT7-MeSAUR1 and pGADT7-MeSAUR1+pAbAi were used as controls. Transformed cells were grown on SD/-Leu selective medium with300 ng/ml AbA at 30°C for 3 days.

### Bioinformatic Analyses of MeSAUR1

The amino acid sequences of SAUR gene families in cassava and *Arabidopsis* were downloaded from Phytozome v12^1^ and NCBI^2^, respectively. A multiple alignment analysis was performed using DNAMAN software. The phylogenetic tree was constructed by MEGA 5.0 using neighbor-joining method (Tamura et al., 2011). Gene or protein accession numbers of cassava and *Arabidopsis* SAUR genes used in this study were listed in **Supplementary Table S1**.

### Expression Profiles Analysis of *MeSAUR1* by Quantitative PCR

Total RNA was extracted by using plant RNeasy extraction kit (Tiangen, CHN) and the first-strand cDNA was reverse-synthesized using the RevertAidTM First-Strand cDNA Synthesis Kit (Fermentas, GER). Quantitative PCR (qPCR) was performed according to Hu’s method (Hu et al., 2016) using Stratagene Mx3000P Real-Time PCR and SYBR^®^ Premix Ex Taq^TM^ (TaKaRa, JPN). The primer pairs of *MeSAUR1* and β*-actin* were list in **Table 1**. The PCR amplification condition used for all reactions was implemented as follows: 95°C for 90 s, 40 cycles of 10 s at 95°C, 15 s at 55°C and 30 s at 72°C. Each sample was amplified in four independent biological replications, and the mean value was used for expression profile analysis. The relative gene expression data were calculated based on the 2^-ΔΔCt^ method (Livak and Schmittgen, 2001). Significant difference analysis between different samples were tested with IBM SPSS Statistics 23 software.

### Subcellular Localization

To confirm the subcelluar location of MeSAUR1, the coding sequence of MeSAUR1 was amplified with V3 primer pair which contained *Bam* HI and *Sal* I sites (**Table 1**). The coding sequence of *MeSAUR1*was introduced into the pCAMBIA1302 vector to generate CaMV35S::MeSAUR1-GFP. Then CaMV35S::MeSAUR1-GFP and pCAMBIA1302 were transformed into *Agrobacterium tumefaciens* and infected onion epidermal cells. The transformed onion epidermal was cultured on MS medium in darkness at 25°C for 24–36 h, and then observation was performed by using confocal microscopy.

### MeSAUR1 Protein Expression and Extraction

The full-length coding sequence of MeSAUR1 was amplified with V2 primer pair (**Table 1**). The PCR product was ligated into the pCold Pros2 vector (TaKaRa, JPN) at the site of *Nde* I/*Sal* I, generating pCold Pros2-MeSAUR1. pCold Pros2-MeSAUR1 was introduced into *Escherichia coli* strain BL21 (DE3) for protein expression. *E. coli* cells containing pCold Pros2-MeSAUR1 were cultured in LB medium supplied with 100 mg/L Ampicillin at 37°C. When the OD_600_ of the culture reaches 0.4–0.8, quickly cool the culture to 15°C in ice water, and then 1.0 mM isopropyl β-D-1-thiogalactopyranoside (IPTG) was added and the cultures were incubated at 15°C, 120 rpm for 24 h. The cells were collected and resuspended in the BugBuster^®^ Protein Extraction Reagent (Novagen, GER) and incubated at 30°C for 1 h. The supernatant was collected and purified with Ni-Charged MagBeads (GenScript, United States). pCold Pros2 was expressed and purified as a control in accordance with the above methods. The purified protein was verified by SDS-PAGE and Western Blotting (Sambrook and Russell, 2001). Tag Anti-ProS2 (Takara, JPN) and Goat Anti-Mouse IgG/HRP (Boster, United States) were the primary and secondary antibodies used in Western Blot, respectively.

### DNA-Protein-Interaction Enzyme-Linked Immunosorbent Assay (DPI-ELISA)

DNA-protein-interaction enzyme-linked immunosorbent assay was according to the method described by Brand et al. (2010). The *MeAGPS1a* promoter biotinylated probe was obtained by PCR with Biotin-*MeAGPS1a* primer pair (**Table 1**). Tag Anti-ProS2 (TaKaRa, JPN) and Goat Anti-Mouse IgG/HRP (Boster, United States) were the primary and secondary antibodies used in DPI-ELISA.

### Dual-Luciferase (Dual-LUC) Assay

The assay was performed according to the method of Hellens et al. (2005). LUC and REN were derived from pGreen II 0800-LUC in this study. The *MeAGPS1a* promoter, obtained with V4 primer pair by PCR, was ligated with LUC through *Spe* I, generating MeAGPS1a pro::LUC. The MeSAUR1 CDS was fused with CaMV35 promoter through *Sal* I and ligated with MeAGPS1a pro::LUC to generate CaMV35S::MeSAUR1-MeAGPS1a pro::LUC. Two constructed vectors were introduced into *A. tumefaciens* strain BLA4404. The culture was infiltrated into the abaxial side of tobacco leaves when its OD_600_ reached to 0.6–1.0. Total protein was extracted from the infected area after culturing for 3 days. The fluorescent values of LUC and REN were detected according to the steps of the Dual-Luciferase Reporter Assay System (Promega, United States). The value of LUC was normalized to that of REN. Due to the sensitivity of this experiment, each experiment used 16 replicates and retaining the majority of 8–10 repetitions for analysis.

## Results

### Identification of *MeSAUR1* in Cassava

AGPase is the rate-limiting enzyme in plant starch biosynthesis, and it is a heterotetramer, containing two small subunits and two large subunits. The amino acids sequences of *Arabidopsis* AGPase family genes were used to BLAST in cassava genome database. Three genes encoded small subunits and five genes encoded large subunits were found in cassava (**Supplementary Table S2**). The transcript level of *MeAGPS1a* was found to be the highest one in AGPase gene family among all tested tissues, and only the MeAGPS1a protein could interact with the large subunits of cassava AGPase by yeast two-hybrid assay (**Supplementary Figure S1**).

**FIGURE 1:**
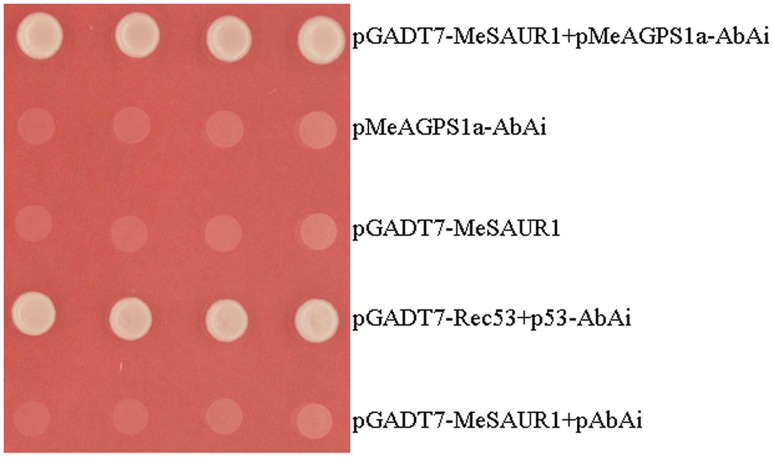
Activation of MeAGPS1a promoter in yeast by MeSAUR1. Yeast cells carried pGADT7-MeSAUR1+pMeAGPS1a-AbAi, pMeAGPS1a-AbAi, pGADT7-Rec53+p53-AbAi, pGADT7-MeSAUR1and pGADT7-MeSAUR1+pAbAi were grown in SD/-Leu selective medium containing 300 ng/ml AbA for 3 days at 30°C.

In order to screen certain transcription factors (TFs) which regulate *MeAGPS1a*, and understand more about the regulation mechanism of starch biosynthesis in cassava storage root, a yeast one-hybrid assay was performed with the *MeAGPS1a* promoter as bait and the storage root cDNA library as prey. Finally, over 300 positive colonies with inserted fragments longer than 750 bp were sequenced after re-checking the primary positive colonies on the same selective medium. Eight of them were annotated as candidate TFs by BLAST analysis. One of them was designated as *MeSAUR1* (Manes.05G149400), coding for an Auxin responsive SAUR protein. The binding specificity between MeSAUR1 and *MeAGPS1a* promoter was further assayed by one to one interaction, only the yeast clones harboring *MeSAUR1* and pAGPS1a-AbAi or positive control could grow on the SD/-Leu selective medium containing 300 ng/ml AbA (**Figure 1**). The results illustrated that MeSAUR1 was able to bind to the *MeAGPS1a* promoter specifically.

### Molecular Characterization of *MeSAUR1*

**FIGURE 2:**
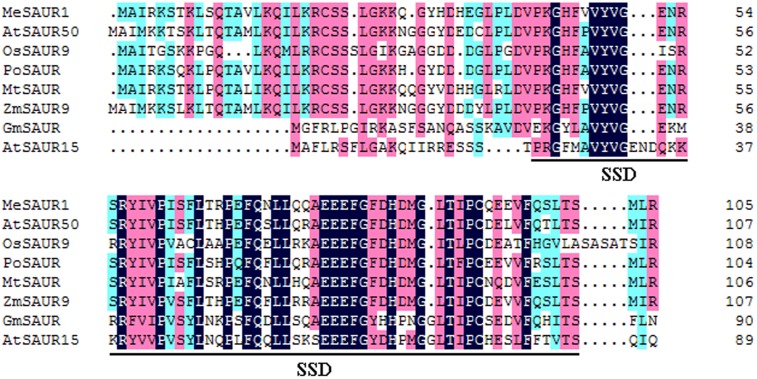
Sequence comparison of MeSAUR1 with related SAUR proteins. The sequences were aligned using DNAMAN6.0. Identical residues are highlighted in dark blue, and biochemically conserved substitutions are highlighted in three gradual darker colors according to the identity. The conserved SAUR-specific domain (SSD) is indicated by bracket. The aligned sequences include MeSAUR1 (Manes.05G149400), AtSAUR50 (At4g34760), AtSAUR15 (At4g38850), OsSAUR9 (Os02g24740), PoSAUR (AFT92005), MtSAUR (XP_013462204), ZmSAUR9 (ACG25834), and GmSAUR (S44175).

**FIGURE 3:**
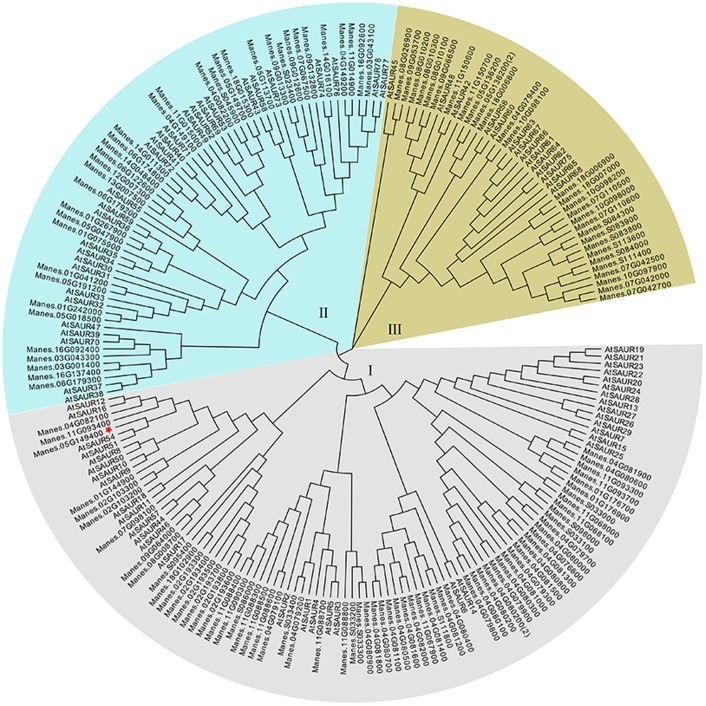
Phylogenetic analysis of 132 MeSAUR and 78 AtSAUR proteins MEGA version 5 from Cluster X2 alignment. The neighbor-joining method was used to construct the tree, the SAUR proteins used in the phylogenetic analysis are retrieved from Genebank and Phytozome, and their gene IDs are listed in **Supplementary Table S1**.

The full-length of *MeSAUR1* cDNA is 930 bp long, including 318 bp CDS, 125 bp 5′UTR and 488 bp 3′UTR, without any intron. It encodes a protein of 105 amino acid residues with a predicted molecular mass of 12.04 kD. The deduced protein contains a conserved SAUR-specific domain (SSD, **Figure 2**). The MeSAUR protein family has 132 members in cassava genome, and can be divided into three clades based on a phylogenetic tree. MeSAUR1 is located in the clade I (**Figure 3**). In addition, there are eight auxin responsive elements (ARE: TGTCNN, Mironova et al., 2014) in the promoter of *MeSAUR1*, especially two TGTCNN elements (-37, -128) locate nearby its transcription start site (**Supplementary Table S3**).

### Expression Analysis of *MeSAUR1*

**FIGURE 4:**
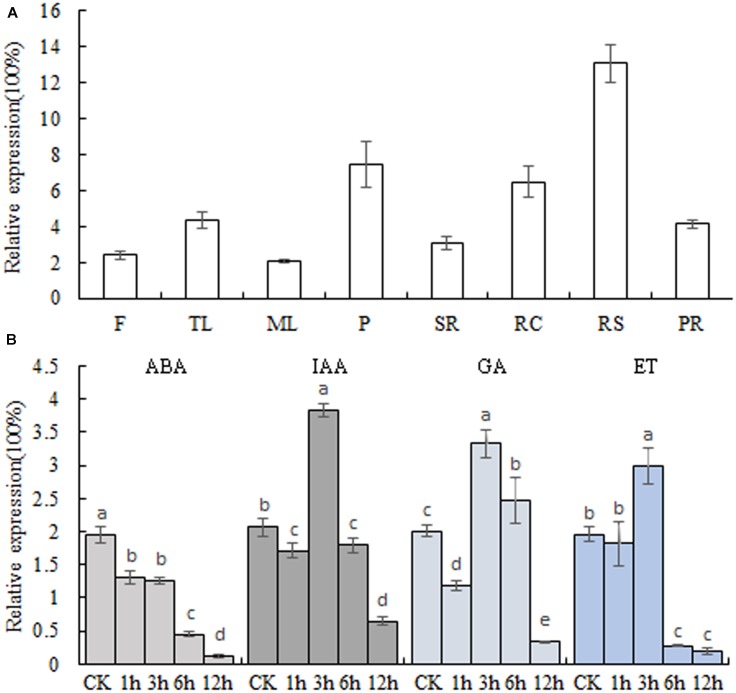
Transcription patterns of *MeSAUR1*. **(A)** Expression pattern of *MeSAUR1* in different tissues of cassava plant. F, flower; TL, tip leaf; ML, mature leaf; P, petiole; SR, stem rind; RC, root cortex; RS, root stele; PR, primal root. **(B)** Transcription profiles of *MeSAUR1* respond to ABA, IAA, GA, and ET. The *y*-axis is the scale of the relative transcript expression. Error bars represent the SD of four technical replicates, the significant difference is assessed by ANOVA at *P* < 0.05. a–e means the significant difference at *p* < 0.05 level.

The transcript levels of *MeSAUR1*in different cassava tissues were measured by qPCR assay, and the results indicated that *MeSAUR1* was highly expressed in root cortex, root stele and petiole, but relatively less expressed in other organs (**Figure 4A**). Furthermore, in order to examine the response to plant hormone for *MeSAUR1*, the cassava tissue cultured seedlings were treated with ABA, IAA, GA, and ET, respectively. The expression level of *MeSAUR1* was reduced by ABA, but induced by IAA, GA, and ET, and reached to its peak all at 3 h after treatment, then remarkably decreased 6 h afterward (**Figure 4B**).

### Sub-cellular Location of MeSAUR1

**FIGURE 5:**
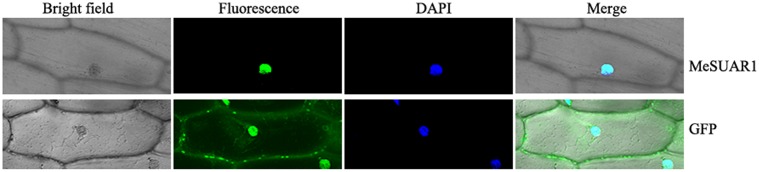
Nuclear localization of MeSAUR1. Top row/Bottom row: the corresponding bright field, fluorescence, merged fluorescence image, and DAPI image of MeSAUR1-GFP/GFP control.

To determine the sub-location of MeSAUR1 protein, the green fluorescent protein (GFP) reporter gene was fused in frame to the N-terminus of MeSAUR1 and transiently expressed into onion epidermal cells by particle bombardment and observed under a fluorescent microscope. The MeSAUR1-GFP fusion protein was located in the nucleus of onion epidermal cell, while the control GFP was dispersed in the onion epidermal cell (**Figure 5**). The result clearly indicated that MeSAUR1 is a nuclear-localized protein.

### MeSAUR1 Can Bind to the *MeAGPS1a* Promoter *In Vitro*

**FIGURE 6:**
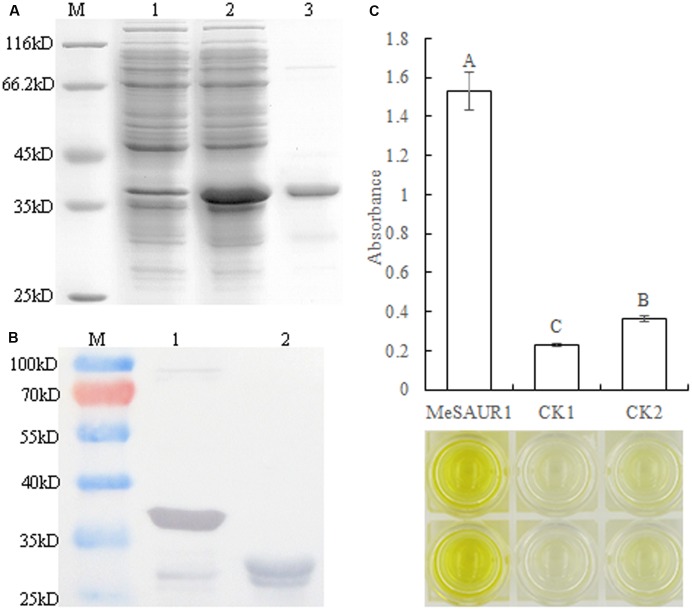
MeSAUR1 binding to the promoter of *MeAGPS1a* as analyzed DPI-ELISA. **(A)** Over-expression of MeSAUR1 in *Escherichia coli*. M: molecular markers, (1) *E. coli* cells harboring pCold Pros2-MeSAUR1 not induced; (2) *E. coli cells* harboring pCold Pros2-MeSAUR1 after 5 h of induction; (3) the purified MeSAUR1 fusion protein by *E. coli cells* harboring pCold Pros2-MeSAUR1 after 5 h of induction. **(B)** Western blot of purified MeSAUR1 fusion protein. M: Molecular markers; (1) MeSAUR1 fusion protein; (2) tagged protein ProS2. **(C)** MeSAUR1 interacts with the *MeAGPS1a* promoter by DPI-ELISA assay. MeSAUR1: double-strand biotinylated *MeAGPS1a* promoter DNA probe + purified MeSAUR1 fusion protein; CK1: double-strand biotinylated *MeAGPS1a* promoter DNA probe + tagged protein ProS2; CK2: purified water + purified MeSAUR1 fusion protein. Error bars represent the SD of four technical replicates, the significant difference is assessed by ANOVA at *P* < 0.01. A–C means the significant difference at *p* < 0.01 level.

In order to determine whether MeSAUR1 interacts with the *MeAGPS1a* promoter *in vitro*, the purified recombinant MeSAUR1 protein was isolated from *E. coli* and its molecular mass was identical to the theoretical value when a 23 kD tagged protein ProS2 was added on (**Figures 6A,B**). Then, we performed DPI-ELISA assay using the recombinant protein and the double-strand biotinylated *MeAGPS1a* promoter DNA probes. The results suggested that MeSAUR1 bound to the *MeAGPS1a* promoter, as the absorbance of MeSAUR1+*MeAGPS1a* promoter was over fourfold higher than those of the two controls (**Figure 6C**). It demonstrated that there was an interaction between MeSAUR1 and *MeAGPs1a* promoter *in vitro*.

### MeSAUR1 Binds to the Region of -400 to -201 in the *MeAGPS1a* Promoter

**FIGURE 7:**
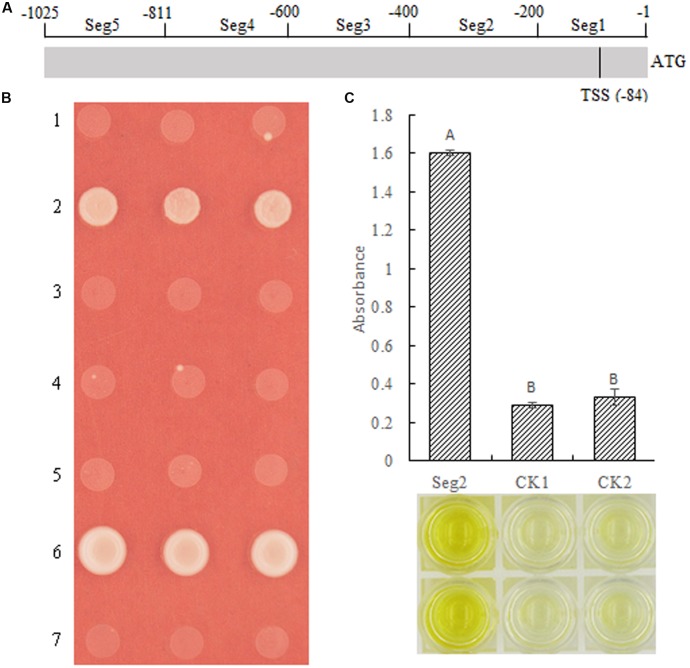
MeSAUR1 binding to the region of –400 to –201 in *MeAGPS1a* promoter. **(A)** The distribution of five segments in the *MeAGPS1a* promoter. TSS: transcription start site. **(B)** Activation of *MeAGPS1a* promoter in yeast by MeSAUR1. Yeast cells carried pGADT7-MeSAUR1 + p(Seg1 to Seg5)-AbAi (1–5), pGADT7-Rec53 + p53-AbAi (6) and pGADT7-MeSAUR1 + pAbAi (7) were grown in SD/-Leu selective medium with 50 ng/ml AbA for 3 days at 30°C; the three dots in each line mean three replications. **(C)** MeSAUR1 interacts with the Seg2 by DPI-ELISA assay. Seg2: double-strand biotinylated Seg2DNA probe + purified MeSAUR1 fusion protein; CK1: double-strand biotinylated Seg2 DNA probe + tagged protein ProS2; CK2: purified water + purified MeSAUR1 fusion protein. Error bars represent the SD of four technical replicates, the significant difference is assessed by ANOVA at *P* < 0.01. A,B means the significant difference at *p* < 0.01 level.

Although we demonstrated that MeSAUR1 was able to interact with the *MeAGPS1a* promoter, its exact binding region was unclear. Therefore, the *MeAGPS1a* promoter was digested into five segments, with each segment about 200 bp, named as Seg1 to Seg5 (**Figure 7A**). Their PCR products were obtained with primer pairs seg1 to seg5 (**Table 1**). These segments were linked to pAbAi vector and transformed into Y1HGold yeast, and the SD/-Ura medium with 50 ng/ml AbA could effectively inhibit the growth of positive transformed Y1HGold yeasts. Then, the vector of pGADT7-AD-MeSUAR1 was transformed into pSeg1-AbAi to pSeg5-AbAi yeast cells, respectively, and pGADT7-AD-MeSUAR1+pSeg2-AbAi yeast cell could grow well in the SD/-Leu selective medium with 50 ng/ml AbA, but other four co-transformed yeast cells could not survive in the same medium (**Figure 7B**). Furthermore, we performed DPI-ELISA assay using the MeSAUR1 recombinant protein and the biotinylated Seg2 DNA probe again (Biotin-seg2, **Table 1**), the results indicated that MeSAUR1 could bind to the region of -400 to -201 in *MeAGPS1a* promoter accurately (**Figure 7C**).

### Activation of the *MeAGPS1a* Promoter by MeSAUR1

**FIGURE 8:**
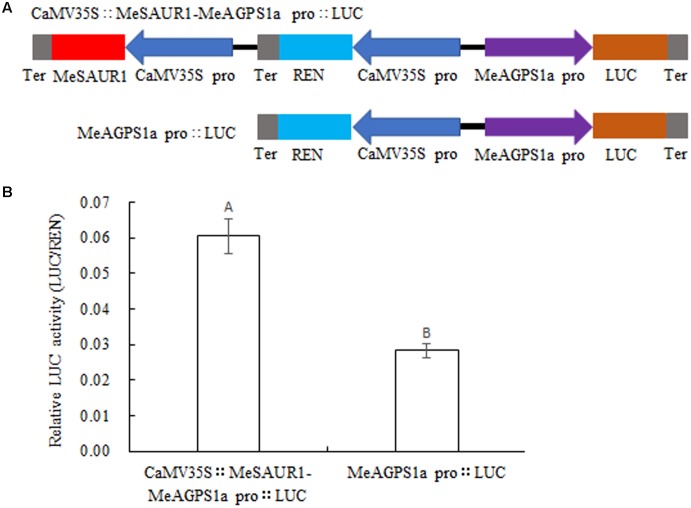
Activation of *MeAGPS1a* promoter in the transient expression system by MeSAUR1. **(A)** Schematic diagrams of the transient expression vectors used in the transient expression analysis. **(B)** Relative LUC activity of vector pCaMV35S::MeSAUR1-MeAGPS1a Pro::LUC and pMeAGPS1a Pro::LUC in tobacco leaves. Error bars represent the SD of nine technical replicates, the significant difference is assessed by ANOVA at *P* < 0.01. A,B means the significant difference at *p* < 0.01 level.

To provide evidence that MeSAUR1 protein regulates the transcription of *MeAGPS1a*, a dual-luciferase reporter assay was introduced. An integrated vector was created which contained 35S::MeSAUR1 and pMeAGPS1a::Luc (**Figure 8A**), then transformed into *Agrobacterium* line LBA4404 for transient expression in tobacco leaves. The relative luciferase activity of pCaMV35S::MeSAUR1-MeAGPS1a pro::LUC was higher than that of pMeAGPS1a pro::LUC (**Figure 8B**), indicating that MeSAUR1 could upregulate the transcript level of *MeAGPS1a* in cassava.

## Discussion

We used the promoter of *MeAGPS1a* as bait and the cDNA expression library of storage root as prey to perform a yeast one-hybrid screening. Eight of the positive colonies were annotated as candidate TFs. This study is focused on MeSAUR1, one of the candidates, which can bind to the -400 to -201 region in *MeAGPS1a* promoter *in vitro* and *in vivo*, and can up-regulate the promoter activity of *MeAGPS1a. SAUR* gene family is auxin responsive factor, and has been reported as functional genes involved in tissue development (Park et al., 2007; Spartz et al., 2012; Kong et al., 2013). MeSAUR1 protein is located in the cell nucleus. Combining all our results, we could conclude that MeSAUR1 is a TF despite the lack of the typical conserved domains of TFs. In addition, further mutation analysis should be used to demonstrate the specific DNA binding site of the SAUR protein.

*MeSAUR1* expression can be induced by the application of exogenous auxin, GA and ET, and repressed by exogenous ABA in cassava. Interestingly, many plant hormone responsive elements are located in the promoter of *MeSAUR1*, e.g., eight AREs, two ERELEE4 (Rawat et al., 2005), three GAREs (Gubler and Jacobsen, 1992) and two ABREs (AATTATTA, **Supplementary Table S3**). It is worth mentioning that the ABRE could interact with ATHB6 protein, and the ATHB6 seemed to represent a negative regulator of ABA signal pathway (Himmelbach et al., 2002). Furthermore, other *SAUR* genes could also be induced or repressed by more than one type of plant hormones, for instance, *AtSAUR36* was induced by auxin and repressed by GA; *AtSAUR*76, 77 and 78 were induced by auxin and ET, and their encoded proteins could interaction with ETR2 and EIN2 as crosstalk points between auxin and ethylene signaling pathway (Stamm and Kumar, 2013; Li et al., 2015). Therefore, MeSAUR1 might be at a crucial crosstalk point in plant hormone signaling pathways, also a mediate for plant hormone to affect starch biosynthesis in cassava.

AGPase is a key enzyme in starch biosynthesis, and over-expressing AGPase genes could increase the amount of its functional proteins, and further improve seed/root starch content and other related traits in cereal and root crops (Sweetlove et al., 1996; Tuncel and Okita, 2013). TF is a protein that can regulate the expression of genes by binding to a specific DNA sequence (Karin, 1990; Latchman, 1997), and many reports have shown that TF can function as transcriptional regulators for starch biosynthesis genes in plant. OsbZIP58 can directly bind to the promoter of six starch biosynthesis genes, *OsAGPL3*, *Wx*, *OsSSIIa*, *OsSBE1*, *OsBEIIb*, and *OsISA2*, and regulate their expression in rice (Wang et al., 2013). Some TFs have shown positive or negative co-expression pattern with starch biosynthesis genes, such as *RSR1*, a member of AP2/ERF family, could up-regulate starch biosynthesis genes and increase starch content in rice endosperm while it gets knocked out (Fu and Xue, 2010). Over-expression of *ZmaNAC36* could up-regulate some starch biosynthesis genes, *AGPL2*, *AGPS2*, *SSI*, *GBSSIIb*, and *SBEI*, in maize endosperm, indicating that ZmaNAC36 is involved in the co-expression of many starch biosynthesis genes, and might play a role in regulating starch biosynthesis (Zhang et al., 2016). MeSAUR1 can bind to the *MeAGPS1a* promoter and activate its promoter activity, suggesting that MeSAUR1 can up-regulate the transcript level of *MeAGPS1a* in cassava. Currently, we are over-expressing or silencing *MeSAUR1* in cassava by a transgenic approach to further investigate and analyze its influence to starch biosynthesis in cassava storage root.

Cassava is an important tuberous root crop with enriched starch in its storage root, and cassava starch is a main staple for over 700 million people in the world (Zhang et al., 2010). However, the molecular mechanism of high starch accumulation in cassava is still unclear. Many studies have been focused on genetic modification of AGPase and granule bound starch synthase (GBSS), few reports are involved in regulatory mechanism of starch biosynthesis in cassava (Ihemere et al., 2006; Zhao et al., 2011; Rolland-Sabaté et al., 2013). A TF, MeSAUR1, identified in this study, can up-regulate the transcript level of *MeAGPS1a*. This is our first step to discover the molecular mechanism of highly efficient starch accumulation in cassava. Subsequently, a series of studies on the TF and cassava starch biosynthesis genes, *MeAGPS1a*, *MeAGPL3*, *MeSus1*, and *GBSSI*, will be carried out. Eventually, we hope to reveal the regulation system, to find the core TFs which can up-regulate the entire starch biosynthesis pathway and to improve starch yield in cassava.

## Conclusion

One cDNA, coding for a small auxin-up RNA protein, named *MeSAUR1*, was isolated from cassava. MeSAUR1 could bind to the promoter in yeast one-hybrid system and *in vitro*, and was located in cell nucleus. The expression level of *MeSAUR1* was high in root cortex, and could be up-regulated under the treatment of IAA, GA, and ET but down-regulated by ABA. MeSAUR1 could activate the promoter of *MeAGPS1a* in tobacco leaves, indicating that it could positively regulate the transcript level of *MeAGPS1a* in cassava.

## Author Contributions

WW and XC designed the research. PM, CLi, YM, ZX, and CLu performed the research. XC and PM wrote the paper. CZ and WW modified the paper. All authors read and approved the final manuscript.

## Conflict of Interest Statement

The authors declare that the research was conducted in the absence of any commercial or financial relationships that could be construed as a potential conflict of interest.

## Footnotes

1 http://phytozome.jgi.doe.gov/pz/portal.html

2 https://www.ncbi.nlm.nih.gov/

## Supplementary Material

**FIGURE S1 |** The interaction between small subunit and large subunit by yeast two-hybrid assay.

**TABLE S1 |** Listed members of 132 MeSAUR and 78 AtSAUR proteins.

**TABLE S2 |** Characteristics of AGPase family members in cassava.

**TABLE S3 |***Cis*-elements distribution in the promoter of MeSAUR1 related to four plant hormones.
