# Supplementary figures and images for: MeSAUR1, Encoded by a Small Auxin-Up RNA Gene, Acts as a Transcription Regulator to Positively Regulate ADP-Glucose Pyrophosphorylase Small Subunit1a Gene in Cassava
Source: Front Plant Sci. 2017 Jul 31;8:1315. doi: 10.3389/fpls.2017.01315 (PMC5534448; doi:10.3389/fpls.2017.01315)

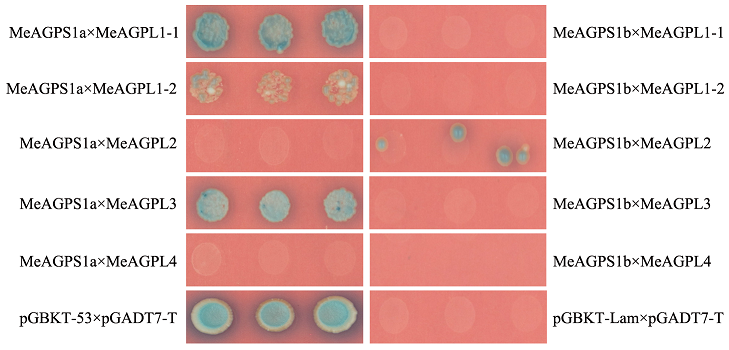

Supplement: Supplementary file 1 [file Image_1.TIF]
