# Supplementary material for: MeSAUR1, Encoded by a Small Auxin-Up RNA Gene, Acts as a Transcription Regulator to Positively Regulate ADP-Glucose Pyrophosphorylase Small Subunit1a Gene in Cassava
Source: Front Plant Sci. 2017 Jul 31;8:1315. doi: 10.3389/fpls.2017.01315 (PMC5534448; doi:10.3389/fpls.2017.01315)
Supplement: Supplementary file 3 [file Table_2.DOCX]

Supplementary Table 2 Characteristics of AGPase family members in cassava

| Isoforms | Transcript name | gDNA size(bp) | Transcript size(bp) | CDS size(bp) | Peptide residues | Theoretical Mw(kDa) | Theoretical pI |
| --- | --- | --- | --- | --- | --- | --- | --- |
| *MeAGPS1a* | cassava4.1_005518m | 4916 | 1893 | 1572 | 523 | 57.33 | 6.69 |
| *MeAGPS1b* | cassava4.1_005446m | 4388 | 1754 | 1581 | 526 | 57.7 | 6.07 |
| *MeAGPS2* | cassava4.1_031078m | 3709 | 1491 | 1491 | 496 | 55.11 | 7.97 |
| *MeAGPL1a* | cassava4.1_005507m | 3299 | 1640 | 1572 | 523 | 58.33 | 9.01 |
| *MeAGPL1b* | cassava4.1_028396m | 3213 | 1569 | 1569 | 522 | 57.87 | 8.73 |
| *MeAGPL2* | cassava4.1_021267m | 3051 | 1431 | 1431 | 476 | 52.56 | 7.88 |
| *MeAGPL3* | cassava4.1_005409m | 6603 | 3659 | 1584 | 527 | 58.55 | 7.57 |
| *MeAGPL4* | cassava4.1_032653m | 4449 | 1587 | 1587 | 528 | 59.1 | 8.68 |
