# Supplementary material for: MeSAUR1, Encoded by a Small Auxin-Up RNA Gene, Acts as a Transcription Regulator to Positively Regulate ADP-Glucose Pyrophosphorylase Small Subunit1a Gene in Cassava
Source: Front Plant Sci. 2017 Jul 31;8:1315. doi: 10.3389/fpls.2017.01315 (PMC5534448; doi:10.3389/fpls.2017.01315)
Supplement: Supplementary file 4 [file Table_3.DOCX]

Supplementary Table 3 Cis-elements distribution in the promoter of *MeSAUR1* related to four plant hormones

| **Cis-element** | **Position/Strand** | **Sequence** | **Expected function** | **Reference** |
| --- | --- | --- | --- | --- |
| ABRE | -1491(+), -1344(-) | AATTATTA | ABA responsive element | Himmelbach et al., 2002 |
| ARE | -37(+), -128(+), -825(+), -1016(+), 1048(+), -1175(+), -1385(+), -1432(+) | TGTCNN | Auxine responsive element | Mironova et al., 2014 |
| ERELEE4 | -223(+), -957(-) | AATTCAAA | Ethylene responsive element | Rawat et al., 2005 |
| GARE | -453(+), -1202(+), -1244(+) | TAACAAA | Gibberellin responsive element | Gubler and Jaccobsen, 1992 |
